# Antioxidant capacity of *Typha angustifolia* extracts and two active flavonoids

**DOI:** 10.1080/13880209.2017.1300818

**Published:** 2017-03-08

**Authors:** Peidong Chen, Yudan Cao, Beihua Bao, Li Zhang, Anwei Ding

**Affiliations:** Jiangsu Collaborative Innovation Center of Chinese Medicinal Resources Industrialization, Nanjing University of Chinese Medicine, Nanjing, China

**Keywords:** *Typha angustifolia*, antioxidant, lipopolysaccharide, human umbilical vein endothelial cells

## Abstract

**Context:** The pollen of *Typha angustifolia* L. (Typhaceae) has been used as a traditional Chinese medicine for improving the microcirculation and promoting wound healing. Flavonoids are the main constituent in the plant, but little is known about the antioxidant activity of the principal constituent of the pollen in detail.

**Objectives:** To assess the antioxidant activities of ethanol and water extracts and two constituents of the pollen.

**Materials and methods:** Plant material (1 g) was extracted by 95% ethanol and water (10 mL × 2, 1 h each), respectively. The extracted activities (0.8–2.6 mg/mL) were measured by DPPH and the reducing activity of ferric chloride (1.7–2.6 mg/mL). Typhaneoside and isorhamnetin-3-*O*-neohesperidoside (I3ON) (2.8–70 μmol/L) were investigated on the relationship between NO, MDA and SOD in HUVECs treated with 100 μg/mL of LPS for 24 h.

**Results:** Nine compounds were identified by UPLC-MS. Ethanol extract showed IC_50_ values in DPPH (39.51 ± 0.72) and Fe^3+^ reducing activity (82.76 ± 13.38), higher than the water extract (50.85 ± 0.74) and (106.33 ± 6.35), respectively. Typhaneoside and I3ON promoted cell proliferation at the respective concentration range of 2.8 to 70 μmol/L (*p* < 0.01). This two compounds decreased MDA (1.91 ± 0.10, 1.80 ± 0.34, *p* < 0.05) and NO levels (14.64 ± 0.08, 13.10 ± 0.88, *p* < 0.01), respectively, and increased SOD level (22.94 ± 2.48, 23.57 ± 2.38, *p* < 0.01) at the concentration of 70 μmol/L compared with LPS group.

**Conclusions:** The constituents from *Typha angustifolia* could be a novel therapeutic strategy for LPS-induced inflammation.

## Introduction

*Typha angustifolia* L. (Typhaceae), a widely distributed aquatic plant, has been used as a traditional Chinese medicine with the abilities to improve the microcirculation, inhibit the immune system (Qin & Sun 2005), induce uterine contractions, treat atherosclerosis and wound healing (Chen et al. 2012), as well as induce the differentiation and stimulate the proliferation of human keratinocytes (Gescher & Deters 2011). The pollen of *Typha angustifolia*, known as Puhuang in China, contains a variety of constituents including flavonoids (Jia et al. 1986; Ishida et al. 1988), stigmasterols (Della Greca et al. 1990), fatty acids (Gallardo-Williams et al. 2002), nucleosides (Tao et al. 2012) and cerebrosides (Tao et al. 2010). According to the pharmacological effects of these compounds, it has been suggested that flavonoids may play a major role as an antioxidant (Bouhlel et al. 2009, 2010). Besides, the constituents in the pollen have diverse biological effects, such as analgesic activity (Ma et al. 2011), inhibition of the contractile activity of isolated uterus (Su et al. 2010), and protection of the human umbilical vein endothelial cells against adrenaline-induced damage (Su et al. 2008).

Lipopolysaccharide (LPS), a bacterial endotoxin, can trigger leukocyte infiltration within the vascular wall and increase vascular permeability (Czermak et al. 1999). Previous studies have demonstrated the relationship between inflammation and oxidants (Yeh et al. 2014). The vascular endothelial system as the largest endocrine organ in the human body plays an important role in maintaining body homeostasis (Li et al. 2013). Free radicals or reactive oxygen species (ROS) induce oxidative stress and enhance oxidative damage after diverse stimuli, which has been confirmed to be an initial event in the development of many diseases such as cardiovascular (Xu & Huang 2007; Liu et al. 2009), inflammatory (Chiu et al. 2013), diabetic (Duarte et al. 2015), neurologic damage (Zhang et al. 2015) and lipid peroxidation of biofilm (Qiu et al. 2014). ROS may be a key factor in the organ injury such as brain and uterus because increased ROS is associated with the decrease in total antioxidant capacity and the significant increase in DNA damage (Kontos 2001; Shu et al. 2013; Singh et al. 2013). Human umbilical vein endothelial cells (HUVECs) could be significant for the elucidation of the antioxidant effects of the pollen. However, little is known about the antioxidant activities of the main constituents of the pollen in detail. In the present study, we assessed the antioxidant activities of ethanol and water extracts of the pollen and the two main constituents in Puhuang using a LPS-induced inflammation model.

## Materials and methods

### Materials and reagents

Pollen of *Typha angustifolia* was collected in Neimenggu province with the voucher specimen number PH-20110801 and identified by Professor Dekang Wu, Department of Medicinal Plants, Nanjing University of Chinese medicine. Typhaneoside and isorhamnetin-3-*O*-neohesperidoside (I3ON) were purchased from China Institute for Control of Pharmaceutical and Biological Products (Beijing, China). 1,1-Diphenyl-2-picrylhydrazyl (DPPH), ferric chloride and LPS were purchased from Sigma-Aldrich Co. (St. Louis, MO). Superoxide dismutase (SOD), malondialdehyde (MDA), and nitric oxide (NO) assay kits were purchased from Nanjing Jiancheng Bioengineering Institute (Nanjing, China). MTT was obtained from Beijing Solarbio Science & Technology Co., Ltd. (Beijing, China). Dimethyl sulfoxide (DMSO) was used as the solvent. All reagents were prepared immediately prior to use.

### UPLC-MS analysis

Pollen (1 g) was extracted twice with 10 times amounts of 70% ethanol (each for 1 h), the extract was centrifuged at 15,000 rpm for 10 min, and then the supernatant was analyzed by UPLC-MS. Analysis was performed with a Waters ACQUITY Ultraperformance LC-MS system coupled with MassLynx. An ACQUITY BEH C_18_ Column (2.1 mm × 100 mm, 1.7 μm) was used. The mobile phase consisted of 0.3% formic acid in water (A) and acetonitrile (B). A total flow rate was 0.2 mL/min and the injection volume 2 μL. The run time was 22 min. Gradient elution was performed starting with 95 → 70% A within 0–5 min; 70 → 69% A within 5–11.5 min; 69 → 35% A within 11.5–18 min; 35 → 95% A within 18–20 min; 95% A within 20–22 min. The column temperature was adjusted to 30 °C. The Mass spectrometer was operated using ESI source in the negative ion mode for detection. Detailed MS parameters were as follows: drying gas (nitrogen) temperature, 350 °C; drying gas flow, 400 L/min; and capillary voltage, 3000 V.

### Determination of DPPH radical scavenging capacity

Total antioxidant capacity (measured as free radical scavenging activity) was evaluated using a stable free radical DPPH following the method described by Germanò et al. (2002) with slight modifications. Briefly, 1 g pollen was extracted with 10 mL 95% ethanol and water, respectively. Each extract (0.05 mL) was added to 0.95 mL of freshly prepared 0.1 mmoL DPPH ethanol solution and incubated in the dark for 30 min at 25 °C. The absorbance was measured at 517 nm. The radical scavenging activity was calculated from absorbance values using the following equation: %inhibition = $\frac{A_{0}-A_{t}}{A_{0}}$ ×100%, where A_0_ was the absorbance of the control (blank, without test compound) and *A_t_* was the absorbance in the presence of the test compound.

### Fe (III) measurement

The Fe (III) measurement was performed by modification of a previously reported method (Sun et al. 2009). Briefly, 1 g pollen sample was extracted by 10 times of 95% ethanol and water, respectively. Each extract was shaken in a shaker bath at 25.0 ± 0.1 °C for 2 h and centrifuged at 6000 rpm. Phenanthroline solution and ferric chloride hexahydrate (FeCl_3_·6H_2_O) solution were prepared in distilled water in a 100 mL ﬂask. Acetate buffer pH 3.5 and 5 was prepared and measured with a calibrated pH meter. An aliquot of each extract was transferred to a 10 mL volumetric ﬂask containing 1 mL of 2 mol/L Fe^3+^ solution and 1 mL of acetate buffer pH 3.5, and then made up to volume with distilled water. The absorbance of each solution was measured at 510 nm against a reagent blank.

### Cell culture and treatment

Human umbilical vein endothelial cells were purchased from Aiyan Biochem. Co. Ltd. (Shanghai, China). The cells were cultured on gelatin coated plastic dishes in RPMI 1640 medium supplemented with 10% FBS, 100 U/mL penicillin, 100 μg/mL streptomycin, 0.135% NaHCO_3_, 15 mM HEPES and 2 mM glutamine. The cultures were incubated at 37 °C in a humidified atmosphere of 5% CO_2_. The medium was changed 24 h after plating and then every 2 days until the cell monolayer reached 80% confluence. The confluent cells were then detached with trypsin-EDTA solution (0.25%: 0.02%). Experiments were conducted using the confluent primary cultures.

### MTT assay

The cells were seeded into 96 well culture plates at the concentration of 1 × 10^5^ cells/well and preincubated for 24 h, followed by incubation for another 24 h with typhaneoside and I3ON (final concentrations of 0.11, 0.56, 2.8, 14, and 70 μmol/L). The cells untreated with compounds served as controls. Cell viability was determined using MTT method. Briefly, 20 μL of MTT solution (5 mg/mL) was added to each well 4 h before the end of incubation. The plates were centrifuged and the untransformed MTT was carefully removed, and extracted with 150 μL of DMSO working solution in each well. The absorbance was measured at the wavelength of 490 nm. Cell survival rate was calculated as: (absorbance of the treated wells)/(absorbance of the control wells) × 100%.

### Cell damage induced by LPS

LPS (100 μg/mL) was added to the confluent HUVECs cell culture and incubated for 24 h at 37 °C in a humidified 5% CO_2_ atmosphere to induce the cell damage. The nuclear morphology was observed under a microscope with excitation at 490 nm.

### Measurement of MDA, NO and SOD

The levels of MDA, NO and SOD were measured according to the manufacturer’s instructions (Nanjing Jiancheng Bioengineering Institute, Nanjing, China). The measurement methods were based on the modifications of previous reports (Liu et al. 2009; Chiu et al. 2013). Briefly, MDA content was measured by the thiobarbituric acid reaction using a commercial kit. A reaction mixture containing 0.2 mL cell lysate and 4.2 mL reaction buffer was boiled for 40 min in water bath, and then cooled down and centrifuged at 4000 rpm for 10 min. The absorbance was read at 532 nm. Nitrite, a stable end product of NO, was then measured by the Griess reaction. Aliquots of samples (100 μL) were mixed with 100 μL of Griess reagent (0.1% N-(1-naphthyl) ethylenediamide dihydrochloride, 1% sulfanilamide in 5% phosphoric acid), followed by spectrophotometric measurement at 550 nm. Nitrite concentrations in the supernatants were determined against a sodium nitrite standard curve. SOD was assessed by the reduction mediated by superoxide anions generated by the xanthine/xanthine oxidase system and monitored at 550 nm.

### Statistical analysis

Statistical analysis was performed using SPSS 16.0 statistical software (SPSS Inc., Chicago, IL). Quantitative data were expressed as mean ± SD. One-way analysis of variance (ANOVA) followed by LSD test was used to analyze data. *p* values of less than 0.05 were considered statistically significant.

## Results

In the present study, DPPH scavenging tests and Fe (III) measurement were used to determine the *in vitro* activities of the ethanol and water extracts of the pollen. Moreover, the antioxidant activities of main constituents in pollen were measured using LPS-induced human umbilical vein endothelial cells.

### UPLC-MS analysis

**Figure 1.**
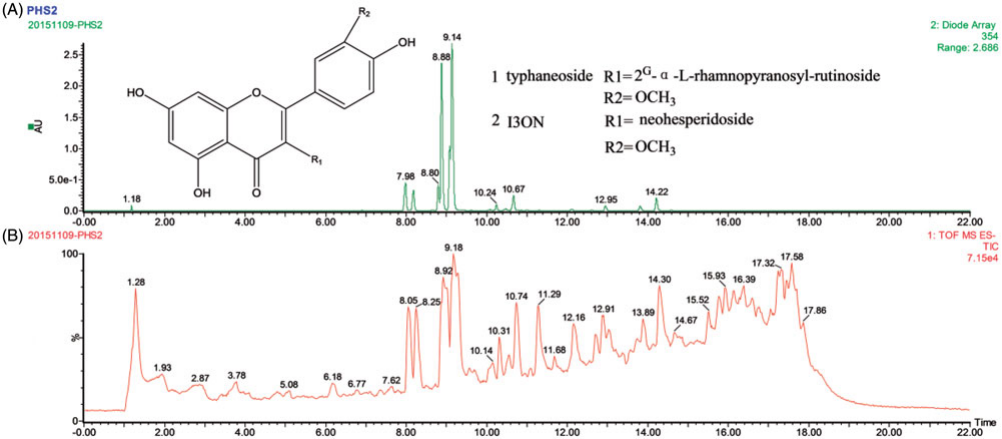
The chromatograph chart at 354 nm (A) and the (−)-ESI total ion current (B) of *Typha angustifolia*.

The main structure group in Puhuang was flavonoids, and the major components were typhaneoside and I3ON as shown in Figure 1. The main constituents in the pollen extracts were analyzed with UPLC-MS based on the LC-MS results as shown in Table 1 and consisted with the results from a previous literature (Tao et al. 2011).

**Table 1.** The identification of flavone compounds in *Typha angustifolia*.

Peak No.	t_R_/min	[M-H]^−^	*λ*_max_(nm) Identification
1	8.05	755,133	255,354 Quercetin -3-*O*-(2^G^-α-L-rhamnosyl)-rutinoside
2	8.25	609,483,363,155	255,354 Quercetin -3-*O*-neohesperidoside
3	8.92	769,269,113	254,354 Typhaneoside
4	9.16	593,329,209,187	264,351 Kaempferol -3-*O*-neohesperidoside
5	9.18	623,593,463	253,354 Isorhamnetin -3-*O*-neohesperidoside
6	12.20	301,249,155,113	255,366 Quercetin
7	12.70	271,177,151	287 Naringenin
8	13.89	285,273,175,155	255,366 Kaempferol
9	4.30	315,300	254,371 Isorhamnetin

### DPPH scavenging tests and Fe (III) measurement

**Figure 2.**
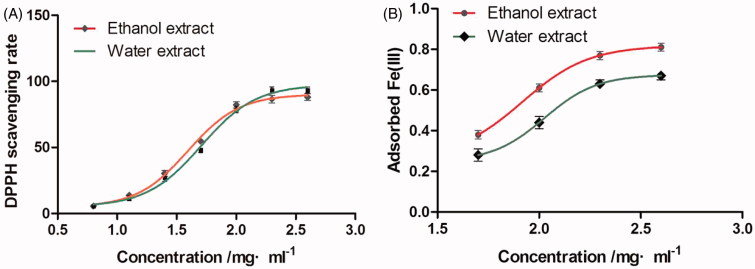
The DPPH scavenging activity (A) and Fe^3+^ absorbance (B) of the extract of *Typha angustifolia*.

The antioxidant capacity evaluation of the pollen extracts was performed by the application of different tests. As reported in Figure 2(A), both ethanol and water extracts showed significant activities in radical scavenging activity tests. The calculated IC_50_ values for the ethanol and water extracts were 39.51 ± 0.72 and 50.85 ± 0.74, respectively. These data correlated with the contents of typhaneoside and I3ON in the two extracts, which were 2.92% and 2.58% in ethanol extract, 2.11% and 2.02% in water extract, respectively. The IC_50_ of the Fe^3+^ reducing activity confirmed the greater effectiveness of ethanol extract (82.76 ± 13.38), which was higher than water extract (106.33 ± 6.35) as shown in Figure 2(B).

### Viability of normal HUVECs

**Figure 3.**
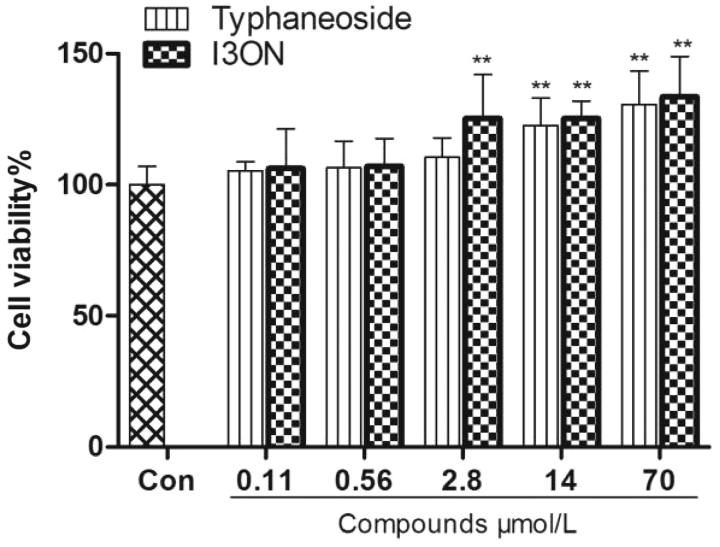
Effect of typhaneoside and I3ON on the activities of normal HUVECs (*n* = 5).

The effects of typhaneoside and I3ON on the normal HUVECs growth were observed by the MTT test at concentrations ranging from 0.11 to 70 μmol/L. After 24 h treatment, the viability of normal cells treated with typhaneoside and I3ON was greater than 90%. The two constituents promoted cell proliferation significantly at the respective concentration range as shown in Figure 3. The cell viability was increased obviously over the concentration range of 2.8 to 70 μmol/L (*p* < 0.01).

### Viability of LPS-induced HUVECs

**Figure 4.**
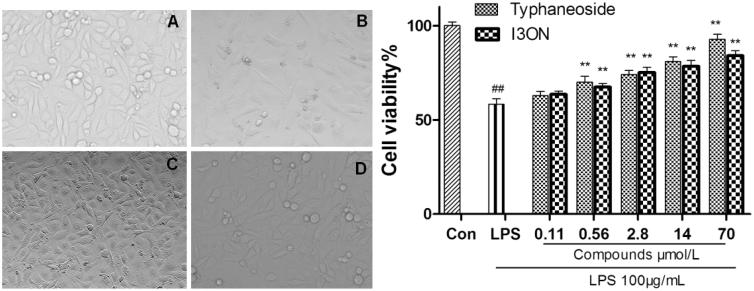
Effect of typhaneoside and I3ON on HUVECs stimulated with LPS. (A) normal control. (B) HUVECs was treated with of LPS (100 μg/mL) stimulation for 24 h. (C) HUVECs induced by LPS was treated with typhaneoside (70 μmol/L). (D) HUVECs induced by LPS was treated with I3ON (70 μmol/L), magnification ×200. Data are expressed as the means ± SD. (standard deviation, *n* = 5). #*p* < 0.05, ##*p* < 0.01 vs. sham control; *p < 0.05, **p < 0.01 vs. LPS group. Con: sham control group, LPS: LPS group.

After 24 h treatment with LPS, the cell viability was reduced to 58.35%. In the presence of typhaneoside and I3ON, the cell viability was increased obviously over the concentration range of 0.56 to 70 μmol/L (*p* < 0.01). Both flavonoid constituents had no effect on cell growth as observed by the MTT test at concentration of 0.11 μmol/L. The cell viability is illustrated in Figure 4. Microscopic examination also confirmed the two constituents promoted significantly the HUVECs cell proliferation which had been inhibited by LPS. The HUVECs cells were flat and polygonal with a typical cobblestone or paving stone mosaic-like arrangement, as shown in Figure 4(A). After 24 h treatment with LPS, the cells presented oval-shape and the number of cells decreased significantly as shown in Figure 4(B). Both typhaneoside and I3ON improved the cell viability distinctly as shown in Figure 4(C,D).

### Measurement of MDA, NO and SOD

**Figure 5.**
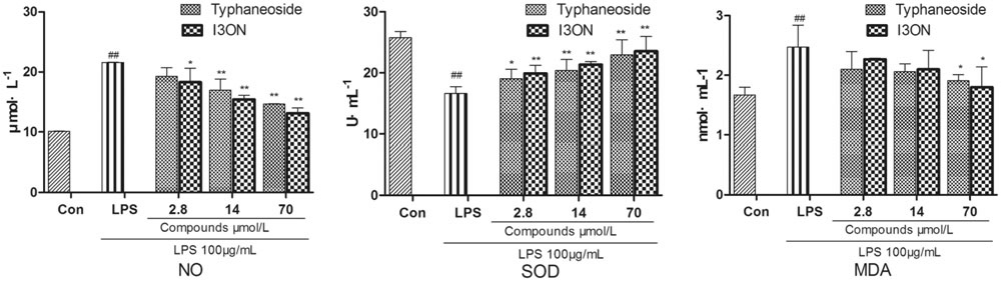
NO, SOD and MDA levels in all groups on HUVECs injury induced by LPS. Data are expressed as the means ± SD (*n* = 5). #*p* < 0.05, ##*p* < 0.01 vs. sham control; *p < 0.05, **p < 0.01 vs. LPS group. Con: sham control group, LPS: LPS group.

NO and MDA levels were found to be increased in the control groups compared to sham control group (*p* < 0.01), and the level of SOD decreased significantly (*p* < 0.01). Elevations of NO and MDA levels in the LPS group demonstrated the validity of the experimental endotoxemia model in HUVECs cultures induced by LPS. Compared with the LPS group, typhaneoside and I3ON significantly decreased MDA (1.91 ± 0.10, 1.80 ± 0.34, *p* < 0.05) and NO levels (14.64 ± 0.08, 13.10 ± 0.88, *p* < 0.01) respectively, and significantly increased SOD level (22.94 ± 2.48, 23.57 ± 2.38, *p* < 0.01) at the concentration of 70 μmol/L. The results of NO, SOD and MDA levels in all groups are presented in Figure 5.

## Discussion

Flavonoids represent the main compound structure type in the pollen, of which, typhaneoside and I3ON are the major constituents. Our experimental results indicated the higher ability of ethanol extract of the pollen to scavenge free radicals. LPS can initiate inflammation in endothelium. Although the relationship between LPS stimulation and endothelial cell apoptosis and related mechanisms are still unclear (Xing et al. 2012), evidence has indicated that large amounts of NO produced by iNOS react rapidly with the superoxide radical to form the potent oxidant peroxynitrite (Gao et al. 2006). NO is a highly reactive free radical, and excess NO can respond to oxygen free radicals and produce the cytotoxic radical ONOO^−^, which can damage cellular functions (Yao et al. 2010). MDA is a by production of lipid peroxidation induced by excessive ROS and is widely used as a biomarker of oxidative stress. The increased level of MDA is associated with increased levels of oxygen free radicals, which attack polyunsaturated fatty acids in cell membranes and cause lipid peroxidation; however, the enzymatic antioxidant defense system is the natural protector against lipid peroxidation (Al-Saeedi & Hossain 2015). SOD is an essential component of the antioxidant defense system. Inhibition of this protective mechanism results in enhanced sensitivity to free radical induced cellular damage (Yang et al. 2011). In our present study, it has been observed that the two main constituents in the pollen could significantly increase the cell survival rate of LPS treated cells, decrease MDA and NO levels and increase SOD compared with LPS group. Furthermore, the tendency to significant increase in the cell survival rate is apparent at the higher dosage. This might be owing to the low toxicity of the compounds in the pollen. Among the effective constituents, typhaneoside and I3ON are flavonoid glycosides. Researchers have revealed the antioxidant activities of I3ON, quercetin and kaempferol in this plant (Bouhlel et al. 2009, 2010; Ye et al. 2012; Surapaneni & Jainu 2014), but little attention has been paid to typhaneoside. Evidence from the available literature has shown that the antioxidant activity of the flavonoids is related to the number of hydroxyl groups on the B ring, especially the 3′-OH and 4′-OH groups; this observation is attributable to the addition of hydroxyl groups to carbon atoms in the *ortho* position, thereby enhancing the antioxidant capacity (Lago et al. 2014). Although no 3′-hydroxyl group, there is a 3′-methoxyl group contained in typhaneoside and I3ON, which also forms the hydrogen bonds. Additionally, a previous study has demonstrated that glycosylation at position 7-OH or 8-OH, but not 3-OH, could impair the antioxidant effect of flavonoids (Cholbi et al. 1991). Thus, the significant antioxidant effect of the pollen should be owing to the structure characteristics of these compounds in the aquatic plant pollen. In our study, flavonoid glycosides increased cell survival and resulted in decreases in MDA and NO levels as well as increase in SOD level.

## Conclusions

Our results suggested that the ethanol and water extracts of the pollen of *Typha angustifolia* have significant radical scavenging activity and reducing ability in a dose-dependent manner. Typhaneoside and I3ON showed protection against LPS-induced endothelial damage by increasing the cell survival rate, decreasing MDA and NO levels while increasing SOD level. These two constituents from the pollen of *Typha angustifolia* could be a novel therapeutic strategy for LPS-induced inflammation.
